# Influenza surveillance in Middle East, North, East and South Africa: Report of the 8th MENA Influenza Stakeholders Network

**DOI:** 10.1111/irv.12628

**Published:** 2019-02-22

**Authors:** Suleiman Abusrewil, Abdulrahman Algeer, Alanoud Aljifri, Fatima Al Slail, Melissa K. Andrew, Mohamed Awad Tag Eldin, Salah Al Awaidy, Nissaf Ben Alaya, Jalila Ben Khelil, Ghassan Dbaibo, Fawzi Derrar, Omar Elahmer, Nada Ghosn, Guelsah Gabriel, Cindy Grasso, Mohamed Hassan, Siddhivinayak Hirve, Yusuf Kamal Mirza, Yousef Moh'd Rateb, Jalal Nourlil, Marta C. Nunes, Idris Omaima, Oliver Ombeva Malande, Mitra Saadatian‐Elahi, Valentina Sanchez‐Picot, Malik Sk. Mamunur Rahman, Hesham Tarraf, Sibongile Walaza

**Affiliations:** ^1^ Medical school Tripoli university Tripoli Libya; ^2^ Medical services directorate of the armed forces/Ministry of defense Riyadh Saudi Arabia; ^3^ Ministry of Health Riyadh Saudi Arabia; ^4^ Dalhousie University and Canadian Centre for Vaccinology Halifax Canada; ^5^ Ministry of Health Cairo Egypt; ^6^ Ministry of Health Mascate Oman; ^7^ Ministry of Health Tunis Tunisia; ^8^ Hopital Abderrahmene Mami Tunis Tunisia; ^9^ American University of Beirut Beirut Lebanon; ^10^ Institut Pasteur d'Algerie Algiers Algeria; ^11^ National Centre for Disease Control Tripoli Libya; ^12^ Ministry of Public Health Beirut Lebanon; ^13^ Heinrich Pette Institute Leibniz Institute for Experimental Virology Hamburg Germany; ^14^ Fondation Mérieux Lyon France; ^15^ Ministry of Health and Prevention Abou Dabi United Arab Emirates; ^16^ World Health Organisation Geneva Switzerland; ^17^ Agha khan university Karachi Pakistan; ^18^ Ministry of Health Amman Jordan; ^19^ Institut Pasteur du Maroc Casablanca Morocco; ^20^ Faculty of Health Science Department of Science/National Research Foundation: Vaccine Preventable Diseases Unit Medical Research Council: Respiratory and Meningeal Pathogens Research Unit University of the Witwatersrand Johannesburg South Africa; ^21^ Faculty of Medicine Cairo University Cairo Egypt; ^22^ Egerton University Nakuru, Kenya & East Africa Centre for Vaccines and Immunization (ECAVI) Egerton Kenya; ^23^ Edouard Herriot Hospital Lyon France; ^24^ World Health Organization Eastern Mediterranean Regional Office Cairo Egypt; ^25^ National Institute of Communicable Disease (NICD) Johannesburg South Africa

**Keywords:** influenza, MENA‐ISN, Middle East, North, East and South Africa, surveillance, vaccination coverage

## Abstract

The Middle‐East and Africa Influenza Surveillance Network (MENA‐ISN), established in 2014, includes 15 countries at present. Country representatives presented their influenza surveillance programmes, vaccine coverage and influenza control actions achieved, and provided a list of country surveillance/control objectives for the upcoming 3 years. This report details the current situation of influenza surveillance and action plans to move forward in MENA‐ISN countries. Data were presented at the 8th MENA‐ISN meeting, organized by the Mérieux Foundation that was held on 10‐11 April 2018 in Cairo, Egypt. The meeting included MENA‐ISN representatives from 12 countries (Algeria, Egypt, Jordan, Kenya, Lebanon, Libya, Morocco, Pakistan, Saudi Arabia, South Africa, Tunisia and United Arab Emirates) and experts from the Canadian Centre for Vaccinology, and the World Health Organization. Meeting participants concluded that influenza remains a significant threat especially in high‐risk groups (children under‐5, elderly, pregnant women and immunosuppressed individuals) in the MENA‐ISN region. Additional funding and planning are required by member countries to contain this threat. Future meetings will need to focus on creative and innovative ways to inform policy and initiatives for vaccination, surveillance and management of influenza‐related morbidity and mortality especially among the most vulnerable groups of the population.

## 1 INTRODUCTION

The Middle‐East and Africa Influenza Surveillance Network (MENA‐ISN) has been established in 2014 and is hosted by Fondation Mérieux since 2017.^1^ The network, initially composed of eight countries, includes 15 countries at present (Algeria, Egypt, Jordan, Iran, Kenya, Lebanon, Libya, Morocco, Oman, Pakistan, Saudi Arabia, South Africa, Tunisia, Turkey and United Arab Emirates). MENA‐ISN works as a think tank to share evidence‐based information, experience and best practices in order to address challenges in control and prevention of influenza, and to increase partnership and networking between countries and international organizations. The main objectives of MENA‐ISN are the following:

- Improve awareness and knowledge of influenza burden through effective communication, education and training;
- Strengthen evidence base through surveillance and research;
- Reduce the incidence of infection through effective prevention measures;
- Increase influenza vaccine uptake and introduction into the national immunization programmes;
- Develop the economic case for sustainable investment of National Plans.

The purpose of this paper was to report on the current situation of influenza surveillance and action plans to move forward in MENA‐ISN countries. Data were originated from the 8th MENA‐ISN meeting, organized by Mérieux Foundation that was held on 10‐11 April 2018 in Cairo, Egypt. The meeting included MENA‐ISN representatives from 12 countries (Algeria, Egypt, Jordan, Kenya, Lebanon, Libya, Morocco, Pakistan, Saudi Arabia, South Africa, Tunisia and United Arab Emirates) and experts from the Canadian Centre for Vaccinology, and the World Health Organization (WHO).

## 2 INFLUENZA PROGRAMME IN EASTERN MEDITERRANEAN REGION

Outbreaks of A/H5N1 (2006 and 2014), A/H1N1 pandemic (2009) and Middle East Respiratory Syndrome‐Coronavirus (MERS‐CoV, 2012) were the main drivers of increasing influenza surveillance in the Eastern Mediterranean Region (EMR). Overall, 19 EMR countries, namely Afghanistan, Bahrain, Egypt, Iran, Iraq, Jordan, Kuwait, Lebanon, Morocco, Occupied Palestinian Territory, Oman, Pakistan, Qatar, Saudi Arabia, Sudan, Syria, Tunisia, United Arab Emirates, Yemen, have functional influenza surveillance systems and 16 National Influenza Centers (NIC) of the WHO Global Influenza Surveillance and Response System (GISRS), are operative and report to the WHO through Eastern Mediterranean Flu (EMFLU), FluNet or Flu Informed Decision (FluID).^2^ Also, the pandemic preparedness tools are available from the WHO.

The main goal of the EMR influenza programmes is to minimize the burden of this vaccine‐preventable disease by (a) monitoring the trends of influenza and locally circulating virus types/subtypes using epidemiological and virological surveillance system for influenza‐like illness (ILI) and severe acute respiratory infections (SARI); (b) describing influenza burden by performing studies on disease burden estimation; and (c) monitoring programme performance and progress towards the set‐up of policies and programmes to increase seasonal influenza vaccination coverage.

In total, 28 professionals from 15 countries were trained to define influenza baseline and threshold values and five countries are working on publishing the results. Overall, 14 (64%) countries have seasonal influenza vaccination policies, five countries included influenza vaccine in their national immunization programmes, and three countries have policies for vaccinating healthcare providers (HCPs). A significant improvement has also been noticed in the number of high‐risk people receiving seasonal influenza vaccines.

The first scientific conference on acute respiratory infection in EMR (EMARIS) was held in December 2017 (Amman, Jordan) and gathered over 140 participants from 20 countries.^3^ One region‐specific panel session was also held during the international conference on emerging infectious diseases in 2015^4^ (Atlanta, USA).

## 3 COUNTRY SITUATION: ACTIONS ACHIEVED AND FUTURE OBJECTIVES

Kenya and South Africa joined the MENA‐ISN in 2018 and participated in the MENA‐ISN meeting for the first time. A summary of major actions achieved in participating countries is provided in Table 1. ILI and SARI surveillance systems are in place in all countries. While some progresses have been achieved, the number of available vaccine doses as well as vaccine coverage remains low. Social mobilization and advocacy campaigns are in place in almost all MENA‐ISN countries.

**Table 1:** Progress achieved in MENA‐ISN countries

Country	Surveillance	Vaccination	Social mobilization	Advocacy and policy
Algeria	6 sentinel sites 1 sentinel for SARI WHO/NIC for virological surveillance National influenza communication plan since 2017	2 500 000 vaccine doses in 2018. Free of charge for risk groups including HCPs	Press conference of Ministry of Health TV/radio spot during vaccination campaign Logo of influenza vaccine on TV screen during news at prime time	Medical societies vaccine recommendations Task force Team with Influenza in agenda
Egypt	SARI and ILI surveillance system 2 NICs for virological surveillance	400 000 vaccine doses	TV programmes, media	Medical societies meetings
Jordan	ILI, SARI sentinel networks and event‐based networks Monitoring of circulating viruses			Political commitment Simple reporting and support of WHO, CDC
Kenya	Improve rapid response during outbreaks Customize and translate WHO recommended tools for surveillance	Improve vaccine uptake and coverage—especially starting with common/potential outbreak areas	Health education and promotion Tailored communication messages	Influenza vaccination policy realized in 2013
Lebanon	12 SARI sentinel surveillance sites			
Libya	Good infrastructure, human resources, laboratory capacities	Vaccine free of charge 1 000 000 doses in 2017 Target children <5 years old in 2018	Media support Health education campaigns	National advisory committee on influenza control established on 2014, official recommendation for influenza vaccination in 2013
Morocco	8 regional laboratories for virus identification	80% of vaccine's price refunded by insurance companies	Media support	Official recommendation for influenza vaccination in 2017
Pakistan	Sentinel‐based influenza surveillance network			Local influenza stakeholder network meeting in October 2017
South Africa	ILI and SARI surveillance, respiratory consultation, influenza‐associated mortality and virological surveillance Viral Watch” ongoing for 25 years [McAnerney et al. 2012]: 97 outpatient departments in 2017	Groups recommended for influenza vaccine identified	Vaccine history collected since 2005	National influenza policy published in 2017 Burden estimates data, guide prioritization for influenza vaccination, and influenza policy and guidelines in place
Tunisia	113 and 6 event‐based sentinel sites for ILI and SARI, respectively Pandemic influenza preparedness and response plan and National Observatory of New and Emerging Diseases since 2009	300 000 doses of influenza vaccine each season to cover high‐risk groups (370 during 2017‐2018 season) Modelling of seasonal influenza	Press conference and regular press release Health education (general population, school, HCPs) Vaccine awareness campaign	Regular training on influenza surveillance, diagnosis and treatment Monthly bulletin on seasonal influenza, WHO weekly report, InPRIS project in collaboration with CDC and WHO Tunisia Workshop on EMFLU with the support of WHO Mena
United Arab Emirates	ILI and SARI surveillance networks (6 hospitals and 11 public health centres) set‐up in 2016 NIC designated Development of national influenza surveillance protocol with EMRO support			National task force to develop annual campaigns to raise awareness and improve vaccine uptake

NIC, National Influenza Centre; MoH, Ministry of Health; NIH, National Institute for Health; PHC, Public Health Centres; HCPs, Health Care Providers.

Future objectives for participating countries are summarized in Table 2. All countries aim at strengthening their surveillance by identifying the needs and by adding detection and monitoring of other novel or emerging respiratory viruses when feasible. Participants also highlighted the need to support the WHO initiative in building laboratory capacity and surveillance in the MENA region and urge the governments to give high priority to the establishment and continued support for influenza surveillance systems.

**Table 2:** Action plan in MENA‐ISN countries

Country	Surveillance	Vaccination	Social mobilization	Advocacy and policy
Algeria	Global Influenza Hospital Surveillance Network (GIHSN), the Global Respiratory Hospitalizations Influenza Proportion Positive (GRIPP)/CDC project		Communication of evidence‐based accurate information through media	Increase awareness, engagement and commitment of HCPs Get the MoH and the media involved
Egypt		Include influenza vaccination in a suitable priority Increase vaccine coverage in HCPs	Increase awareness on influenza complications, safety & efficacy of vaccination	Influenza burden studies in high‐risk groups Study on influenza disease outbreaks in pregnant women
Jordan	Strengthen Surveillance and early warning system	Targeted seasonal influenza vaccination programme, strategic stockpiling of antiviral drugs and personal protection equipment Improve vaccine coverage in HCPs and high‐risk groups	Training, health education and communication	
Kenya	Improve rapid response during outbreaks Customize and translate WHO recommended tools for surveillance	Improve vaccine uptake and coverage	Health education and promotion Tailored communication messages	Training for HCPs
Lebanon	ILI surveillance in both private and medical centres		Preventive and control actions	
Libya	Strengthen ILI surveillance in public centres action plan	Increased vaccination coverage to include children under 5 years old	Increase awareness of influenza vaccine among health workers	Develop research capacity and strengthen multisectoral coordination
Morocco	Influenza and SARI surveillance workshops for Hospital Staff Virological diagnostic capacities Update of virological surveillance guide	Influenza vaccine efficacy study among HCPs Increase the vaccine coverage rate among HCPs, elderly and diabetics	Design educational material with key messages on influenza Produce TV/radio spot during flu vaccination campaign	Produce communication tools and organize seminars for HCPs during immunization campaign
Pakistan	Incorporate SARI surveillance Influenza‐related morbidity and mortality estimates Prospective targeted surveillance at point of entry Include other respiratory pathogens Disease burden studies	Provide/increase official recommendations for influenza vaccination Raise vaccination coverage among high risk Increase funding for vaccination by government	Maternal education for childhood influenza Appropriate media messages	Country‐specific guidelines for preventive interventions in high‐risk groups Inclusion of influenza‐associated SARI in National Priority disease list Education of general practitioners
Tunisia	Thresholds for seasonal influenza surveillance Implementation of electronic information system Improvement of SARI surveillance	Increase number of available doses of vaccine Increase coverage of pregnant woman National survey on vaccine effectiveness		Vaccine acceptance by HCPs
United Arab Emirates	Enhance epidemiological and laboratory surveillance	Incorporate influenza vaccination in clinical management guidelines		Develop research capacity and enhance multisectoral coordination and collaboration

QIV, Quadrivalent influenza vaccine.

Regarding influenza vaccines, developing long‐term plans for the use of seasonal influenza vaccines at least among high‐risk groups, providing/increasing funding for vaccination by governments and setting‐up vaccination campaigns were reported as the main actions to increase vaccine uptake.

In addition to the lack of adequate funding to procure influenza vaccines, poor awareness in the public and HCPs about influenza/vaccine, vaccine hesitancy, absence of clear messages and social mobilization could also contribute to low vaccination coverage in MENA region. Several programmes on education of the community and HCPs are currently ongoing, but more still needs to be done.

Finally, influenza burden (ie morbidity, mortality, loss of working hours) is not well described. More research to clearly estimate the economic burden of influenza and cost of “inaction,” to generate new evidence on influenza virus circulation and disease burden can serve as the basis to increase uptake and coverage of seasonal influenza vaccines and to guide measures for pandemic influenza.

The MENA‐ISN roadmap is now available to help achieving the network's goals.

## 4 DISCUSSION AND CONCLUDING REMARKS

Emerging infectious diseases are a major public health problem in the EMR region. Besides, more than half of the 22 countries of the EMR are affected by acute or protracted complex emergencies.

To increase influenza vaccination coverage, MENA‐ISN countries could start by targeting HCPs and high‐risk individuals and scale up to other groups and ultimately to the whole population.

Pregnant women are considered by the WHO as the main high‐risk group during pandemics. Maternal immunization can reduce the risk of post‐natal infections in the infants.^5^ Currently, two studies among pregnant women are ongoing in Tunisia and Saudi Arabia. One aims at using antibodies in the umbilical cord of women who received vaccination during pregnancy as a marker of response to influenza virus vaccination in pregnant women. Two other studies on the critical window of vaccination during pregnancy and a prospective observational cohort study to determine the burden of influenza disease in pregnant women and in the newborns in the MENA region are under examination by the expert group.

Elderly individuals are also at high risk of influenza‐related hospitalization and death.^6^, ^7^ Frailty is defined as “a state of increased vulnerability to poor resolution of homoeostasis after a stressor event”, which increases the risk of adverse outcomes,^8^ and represents a new way to think about vulnerability to influenza^9^, ^10^ that can be used in the MENA region for at‐risk individuals. For example, frailty has been studied in influenza surveillance in Canada, where a frailty index based on a Comprehensive Geriatric Assessment has been found to impact estimates of influenza vaccine effectiveness and outcomes of hospitalization with influenza illness.^9^ Given that frailty can be measured in different ways, work is ongoing to identify and validate brief measures of frailty such as the Clinical Frailty Scale which may be more readily implemented in resource‐diverse settings.^11^

Pilgrims to Saudi Arabia are a specific high‐risk group for influenza infection and transmission in the MENA region. Influenza vaccination is recommended since 2005 but few studies assessed influenza vaccine effectiveness in pilgrims and its impact on other respiratory illnesses.^12^, ^13^ Arising from discussions at the 8th MENA‐ISN meeting, a multicentre hospital‐based study is under investigation by MENA‐ISN participating countries. A questionnaire will be prepared to collect data on vaccination status of pilgrims, SARI symptoms and other factors that could impact vaccine effectiveness.

Currently, trivalent inactivated influenza vaccine is the main vaccine in use in the majority of MENA countries. Considering different vaccine products, which could be targeted to different high‐risk groups (eg young children, immunosuppressed or elderly people) in MENA region, has the potential to provide greater effectiveness that would also help in combating the phenomenon of vaccine hesitancy. Introduction of quadrivalent vaccine covering both influenza B lineages could be beneficial since both lineages have been detected in MENA countries. Given that elderly people tend to have diminished immune responses compared with younger adults,^14^ increasing vaccination rates to protect elderly people, potentially using vaccine products which lead to more vigorous immune response, is also fundamental. Recently, several influenza vaccines that could have a better action have been tested in elderly people. The use of adjuvanted sub‐unit vaccine (MF59) has been proven to be effective in reducing several influenza‐related outcomes among the elderly, especially hospitalizations due to influenza‐related complications.^15^ Compared with standard‐dose influenza vaccine, high‐dose vaccine was also found to reduce hospital admission with respiratory illness^16^, ^17^, ^18^ and to be cost‐saving.^19^, ^20^ Finally, a recombinant influenza vaccine has been shown to provide a better protection in older adults than the standard quadrivalent, inactivated influenza vaccine.^21^ At present, the availability of these newer vaccine products is limited in many settings, and where available, they may be cost‐prohibitive. With this in mind, implementing a realistic vaccination strategy with available product in order to improve influenza vaccine coverage should be considered as the first‐line strategy with whatever influenza vaccine available in a given country.

In summary, despite enhanced influenza surveillance systems, a higher number of NICs that actively perform influenza virus typing and more available information on influenza burden in the region, the current situation is not satisfactory. Key challenges for the future are sustainability, good quality data and more private‐academic collaborations under a common scientific umbrella. A unified group of committed national experts, that is MENA‐ISN with joint multisectoral and multidisciplinary efforts, will help to get further than individual efforts. The network can be the active advocate for acceptance of vaccination by HCPs and the public, by bringing key local stockholders together, explaining benefits of seasonal influenza vaccination and addressing misinformation. Dissemination of surveillance and disease burden data through publications and effective communication tools will help to push health authorities in favour of official recommendations for influenza vaccination.

## CONFLICT OF INTEREST

The authors declare no conflict of interest to report.
